# Search for mesophotic octocorals (Cnidaria, Anthozoa) and their phylogeny. II. A new zooxanthellate species from Eilat, northern Red Sea

**DOI:** 10.3897/zookeys.676.12751

**Published:** 2017-05-23

**Authors:** Yehuda Benayahu, Catherine S. McFadden, Erez Shoham, Leen P. van Ofwegen

**Affiliations:** 1 School of Zoology, George S. Wise Faculty of Life Sciences, Tel Aviv University, Ramat Aviv, 69978, Israel; 2 Department of Biology, Harvey Mudd College, Claremont, CA 91711-5990, USA; 3 Department of Marine Zoology, Naturalis Biodiversity Centre, P.O. Box 9517, 2300 RA Leiden, the Netherlands

**Keywords:** Octocorallia, taxonomy, new species, mesophotic coral ecosystem, Eilat, Red Sea

## Abstract

An octocoral survey conducted in the mesophotic coral ecosystem (MCE) of Eilat (Gulf of Aqaba, northern Red Sea) yielded a new species of the speciose reef-dwelling genus *Sinularia*. It features encrusting colony morphology with a thin, funnel-shaped polypary. *Sinularia mesophotica* **sp. n.** (family Alcyoniidae) is described and compared to the other congeners with similar morphology. Both the morphological and molecular examination justified the establishment of the new species, also assigning it to a new genetic clade within *Sinularia*. The results highlight its unique phylogenetic position within the genus, and this is the first described species of a mesophotic zooxanthellate octocoral.

## Introduction

The taxonomy of the northern Red Sea octocorals has been quite extensively studied, albeit mostly confined to the reefs above 30 m (references in Shoham and Benayahu 2017). Studies have demonstrated a high octocoral richness in the Red Sea, revealing new taxa and new zoogeographical records (e.g., Verseveldt and Benayahu 1978, 1983; Haverkort-Yeh et al. 2013; Ofwegen et al. 2013, 2016). Octocorals of the mesophotic coral ecosystems (MCEs), in contrast, have remained little studied (Shoham and Benayahu 2017). To date, the only newly-described mesophotic Red Sea octocoral is the azooxanthellate *Scleronephthya lewinsohni* Verseveldt and Benayahu, 1978 of the family Nephtheidae, discovered at a depth of 55–82 m. Recently, Shoham and Benayahu (2017) recorded a higher species richness and higher number of species in Eilat’s upper MCEs (30-45 m) compared to the shallower reefs there. The latter study also revealed an almost exclusive dominance of zooxanthellate octocorals in the upper MCE. Following an octocoral survey conducted in Eilat’s MCEs, we describe here a new species of the genus *Sinularia*.

## Materials and methods

Samples were collected by ROV (ECA H800) operated by the Sam Rothberg R/V of the Interuniversity Institute for Marine Sciences in Eilat. *In-situ* photography was carried out using a low light black and white camera VS300 (Eca Robotics) and 1CAM Alpha HD camera (SubCimaging). Samples were obtained using the ROV arm. Colony fragments were removed and preserved in 100% ethanol for molecular work. The original samples were placed in 70% ethanol for taxonomic identification, for which sclerites from different parts of the colonies (polyp, polypary surface and interior, base surface and interior) were obtained by dissolving the tissues in 10% sodium hypochlorite, followed by rinsing in fresh water. Sclerites were then prepared for scanning electron microscopy as follows: rinsed with double-distilled water; dried at room temperature, coated with gold-palladium; and examined with a SEM Jeol 6480LV electron microscope and at high vacuum under an environmental scanning electron microscope (ESEM, JSM-6700 Field Emission Scanning Electron Microscope, operated at 10 kV). Wet preparations of tissue smears were examined under a light microscope (X 200) in order to verify presence of symbiotic algae (zooxanthellae). Material studied is deposited at the Steinhardt Museum of Natural History, National Center for Biodiversity Studies, Tel Aviv University, Israel (ZMTAU) and Naturalis Biodiversity Center, formerly Rijksmuseum van Natuurlijke Historie, Leiden, the Netherlands (RMNH).

### Molecular phylogenetic analyses

DNA was extracted from the EtOH-preserved samples, and two mitochondrial gene regions (*mtMutS*, *igr1* + *COI*) were sequenced using previously published primers and protocols (McFadden et al. 2011). Sequences were aligned to a dataset that included published sequences from 143 specimens representing >85 nominal species of *Sinularia* (McFadden et al. 2009, 2014; Benayahu et al. 2013). Maximum likelihood analyses of each gene region separately as well as the concatenated sequence were conducted using PhyML (Guindon et al. 2003) with 100 bootstrap replicates. A GTR+I+G model of evolution was substituted for the best-fit TVM+I+G model selected using ModelTest 3.0 (Posada and Crandall 1998) but not available in PhyML. MEGA v. 5 (Tamura et al. 2011) was used to calculate pairwise genetic distance values (Kimura 2-parameter) between sequences. New sequences have been deposited in GenBank (KY971524–KY971525), and alignments and treefiles in TreeBase (http://purl.org/phylo/treebase/phylows/study/TB2:S20934).

## Results

### Systematic description

#### Order Alcyonacea Lamouroux, 1912

##### Family Alcyoniidae Lamouroux, 1912

###### Genus *Sinularia* May, 1898

####### Sinularia mesophotica sp. n.

Taxon classificationAnimaliaAlcyonaceaAlcyoniidae

http://zoobank.org/658B7592-DE2D-4929-AF61-C11FABCF2879

Figs 1 , 2 , 3 , 4

######## Type material examined.

Holotype: ZMTAU Co 37425, Israel, northern Red Sea, Gulf of Aqaba, Eilat, Dekel Beach (29°32'2.49"N, 34°57'44.56"E), 62 m, 31 May 2016, coll. M. Weis; three paratypes: ZMTAU Co 37492 same collection details.

######## Diagnosis.

**Figure 1.**
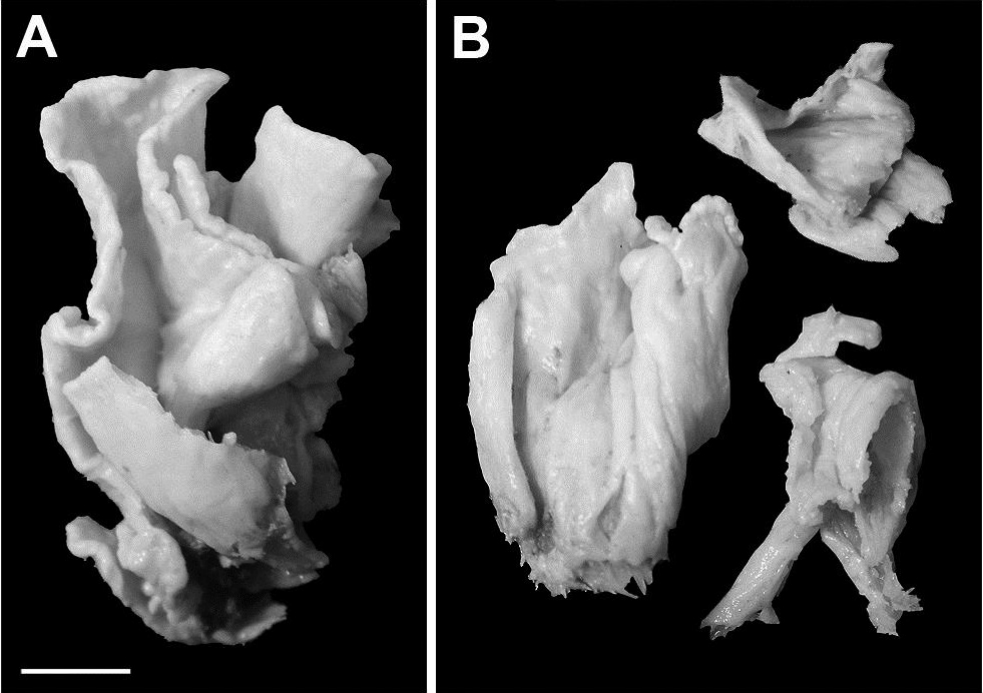
*Sinularia mesophotica* sp. n.; **A** Holotype ZMTAU Co 37425 **B** paratypes ZMTAU Co 37492. Scale bar: 1 cm (**A** also applies to **B**).

**Figure 2.**
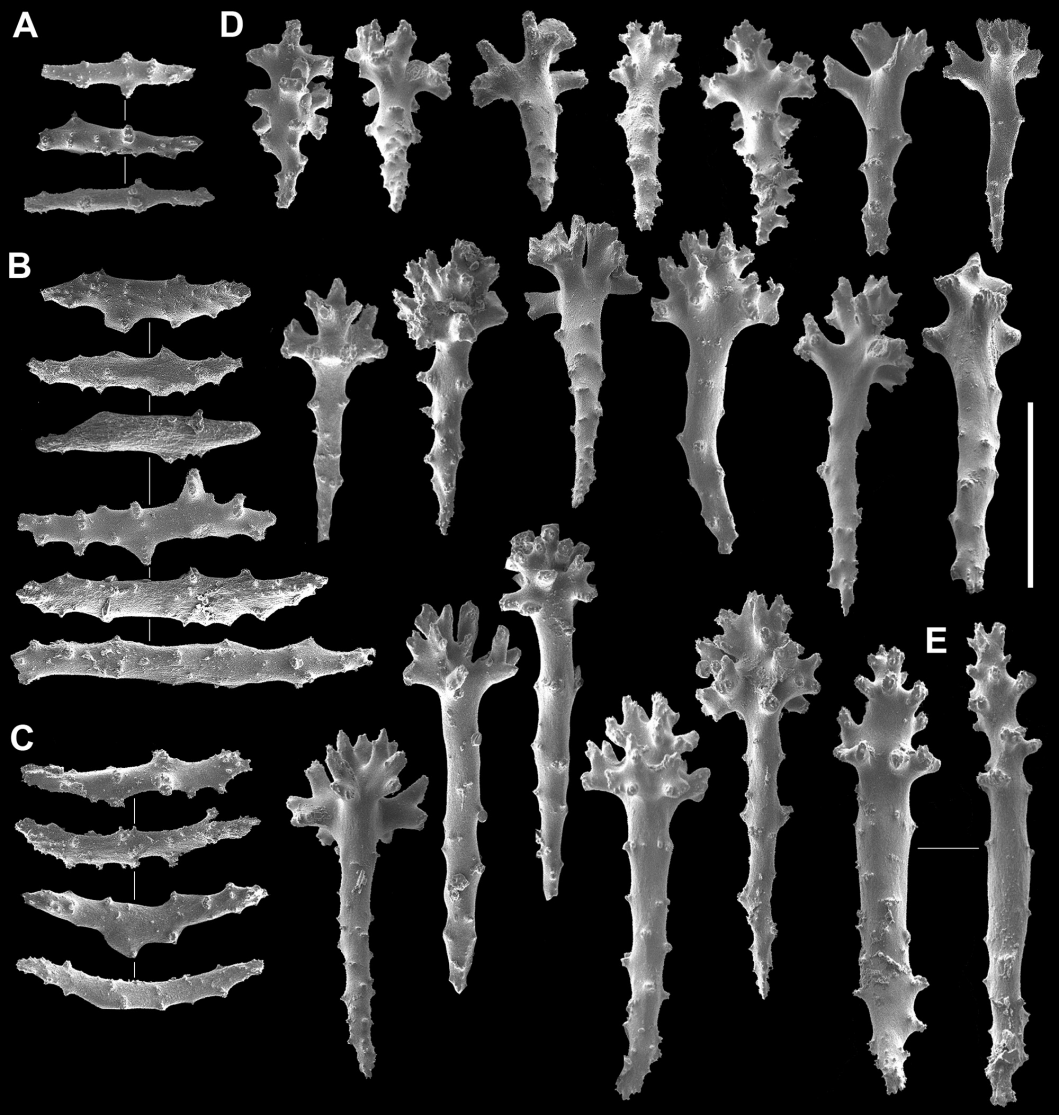
*Sinularia mesophotica* sp. n., holotype ZMTAU Co 37425. Sclerites from the polypary. **A** tentacle rods **B** straight collaret spindles **C** bent collaret spindles **D** clubs **E** larger clubs. Scale bar: 0.10 mm.

**Figure 3.**
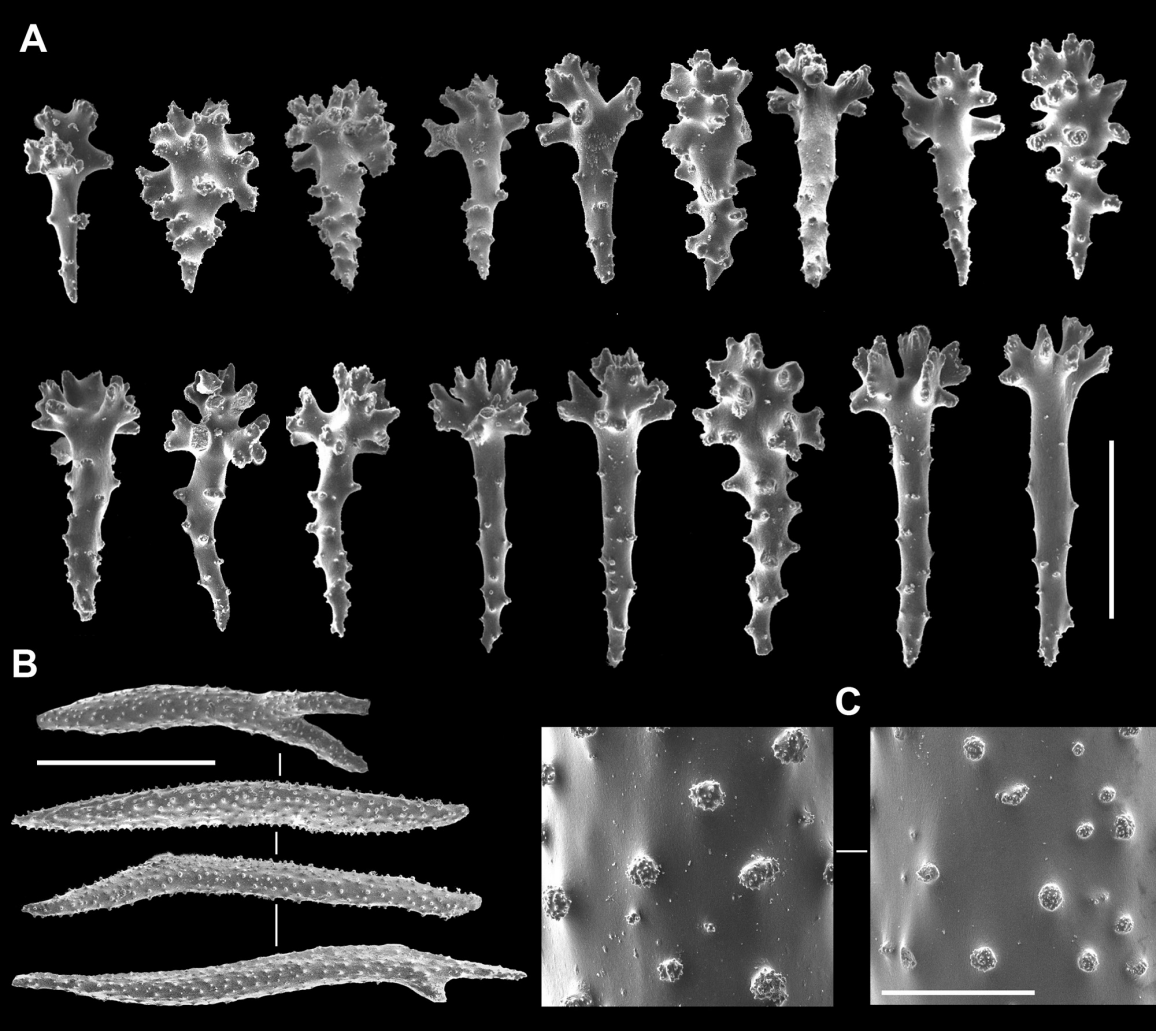
*Sinularia mesophotica* sp. n., holotype ZMTAU Co 37425. Sclerites of the base of colony. **A** clubs of the surface layer **B** spindles of interior **C** tuberculation of the spindles. Scale bars: 0.10 mm (**A, C)**, 1 mm (**B**).

The holotype is part of an encrusting colony with a thin, funnel-shaped polypary, also featuring a curly margin (Fig. 1A). In a side-view its maximum dimensions are 5 × 2.5 cm. Polyps with tentacle rods and collaret sclerites (Fig. 2A–C). Tentacle rods up to 0.10 mm long (Fig. 2A). Collaret consists of almost straight spindles, up to 0.20 mm long (Fig. 2B), and shorter bent ones, up to 0.14 mm long (Fig. 2C). Surface layer of the polypary with clubs (Fig. 2D), some featuring a central wart, while in others it is less discernible, or even absent. Clubs vary from 0.10 mm long to 0.25 mm long, and a few with poorly developed heads attain 0.27 mm (Fig. 2E). Surface layer of the colony base contains clubs up to 0.22 mm; some similar to those of polypary, and others have wide heads (Fig. 3A). Polypary and base interior bear spindles, some branched, up to 3.2 mm long (Fig. 3B), with well-spaced simple tubercles (Fig. 3C).

######## Color.

The ethanol-preserved holotype is beige.

######## Etymology.

The new species name reflects its mesophotic habitat.

######## Living features.

**Figure 4.**
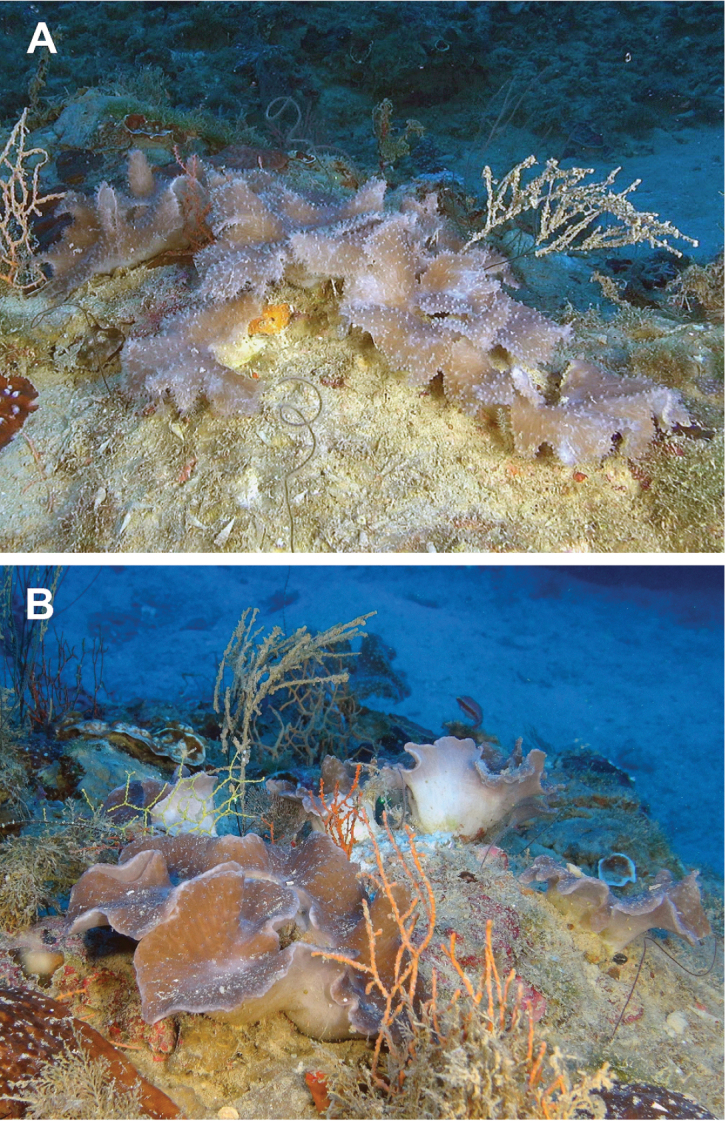
Underwater photographs of *Sinularia mesophotica* sp. n. **A** patch of colonies **B** funnel-shaped morphology of colonies.

Colonies grow as dense patches over reefal-calcareous substrate. Their polypary is flat and horizontally oriented (Fig. 4A) or funnel-shaped (Fig. 4B), with upper part dark-brown, due to dense zooxanthellae as verified by light microscopy, and lower part brighter, almost white. The flexibility and softness of the living colonies was recognized by their movement in the water currents generated by the ROV arm.

######## Variability.

ZMTAU Co 37492 comprises three paratypes, each of them represented by fragments of colonies (Fig. 1B). Their general morphology and sclerites are identical to those of the holotype ZMTAU Co 37425.

### Molecular phylogenetic analyses of *Sinularia mesophotica*

**Figure 5.**
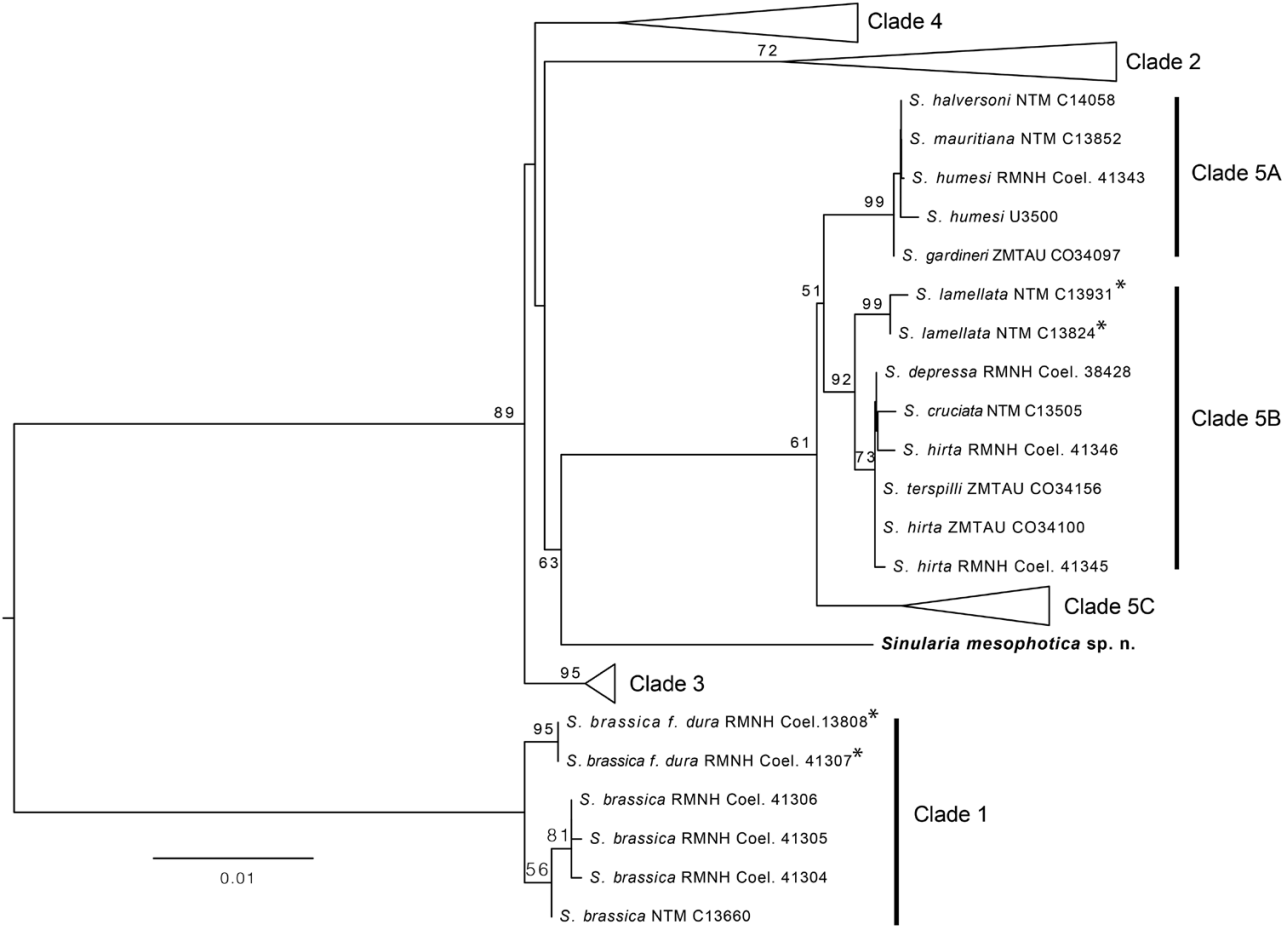
Maximum likelihood tree of concatenated *mtMutS* and *igr1+COI* mitochondrial gene sequences. Clade numbering system follows McFadden et al. (2009); some clades have been collapsed to facilitate readability. Asterisks indicate species with a funnel-shaped morphology similar to that of *Sinularia mesophotica* sp. n. Bootstrap values >50% are indicated adjacent to nodes.

Phylogenetic analyses of *mtMutS*, *igr1 + COI* (not shown) and the concatenated sequence (Fig. 5) all placed *Sinularia mesophotica* n. sp. in a unique position outside the five previously recognized clades of *Sinularia* (McFadden et al. 2009). The mean genetic distance (Kimura 2-parameter) between *S. mesophotica* n. sp. and all other species was 5.7% (s.d. ± 1.0%), comparable to the mean distances between different clades (2.8–7.2%), and much greater than what is typically observed between species within each clade (0.2–2.9%) (McFadden et al. 2009). The *mtMutS* tree (not shown) suggested that *S. mesophotica* n. sp. is a sister taxon to clade 5 (bootstrap support = 78%), but neither *igr1+COI* (not shown) nor the concatenated gene tree resolved the basal relationships among *S. mesophotica* sp. n. and clades 2–5 (Fig. 5).

## Discussion

Prior to the present study two *Sinularia* species have been described as possessing a distinct funnel-shaped polypary. Certain morphologies of *S. brassica* May, 1898, originally assigned to *S. dura* (Pratt 1903), share such morphology (see Benayahu et al. 1998: figs 6, 7, 9, 13, 25). Notably, *S. brassica* features club-sclerites with heads consisting of two or three diverging, wide-toothed prominences, markedly different from those of *S. mesophotica*.

**Figure 6.**
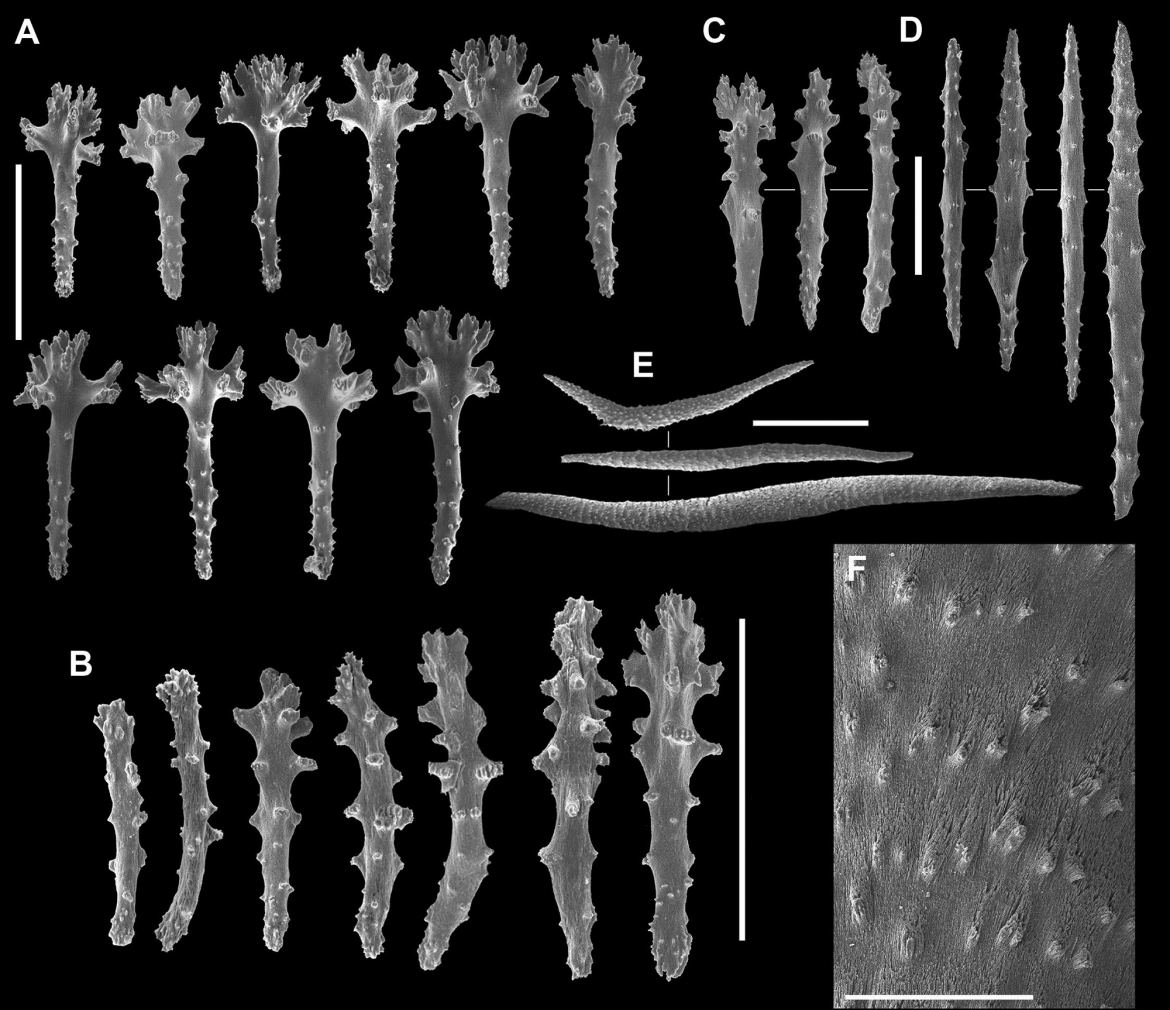
*Sinularia lamellata* RMNH Coel no. 12864. Sclerites of the polypary. **A** clubs of surface layer **B–C** point sclerites **D** spindles from surface **E** spindles from interior **F** tuberculation. Scale bars: 0.10 mm (**A–D, F**), 1 mm (**E**).

**Figure 7.**
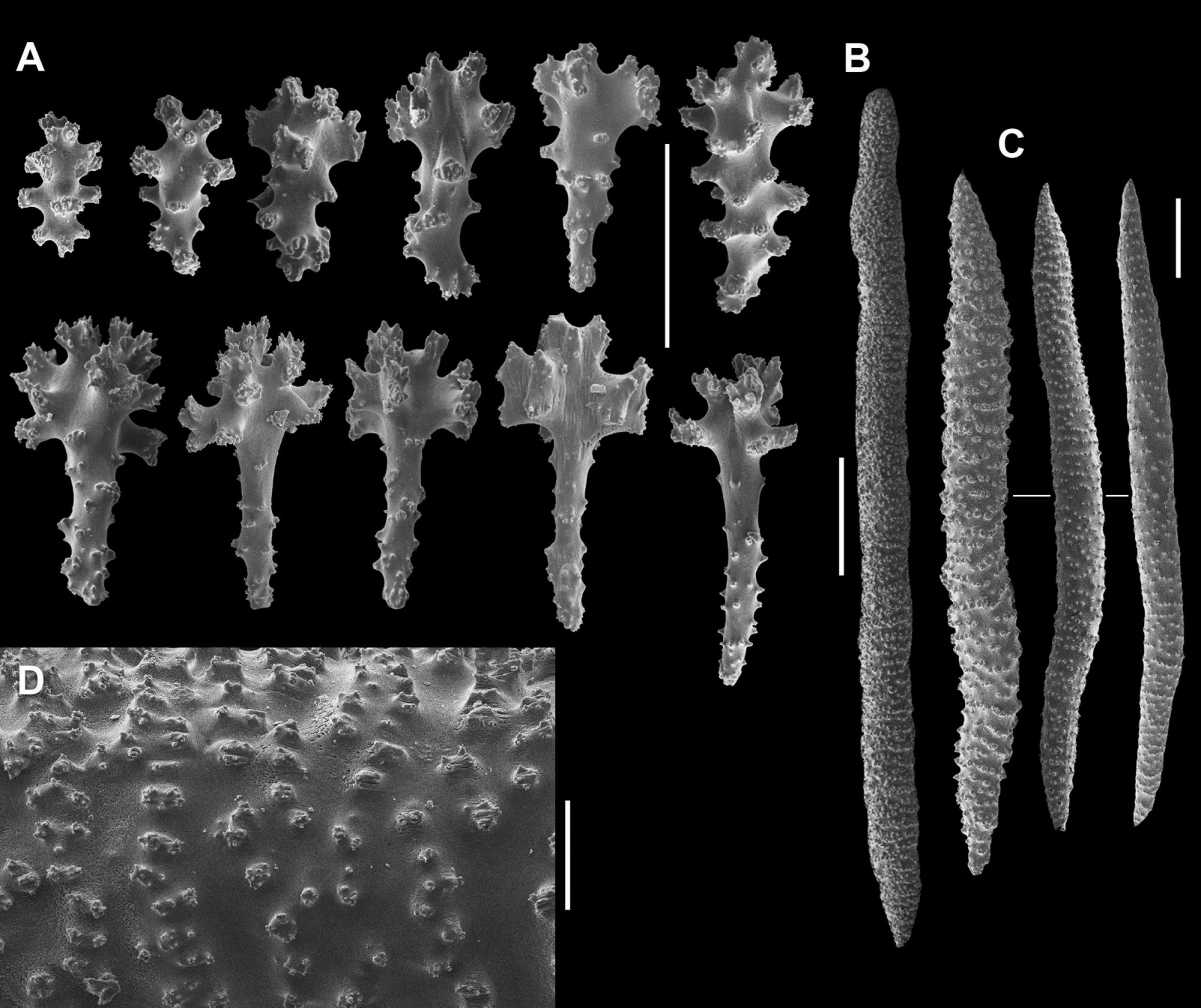
*Sinularia lamellata* RMNH Coel no. 12864. Sclerites of the colony base; **A** clubs of surface layer **B–C** spindles from interior base **D** tuberculation of spindles. Scale bars: 0.10 mm (**A, D**), 1 mm (**B, C**).

The holotype of *Sinularia lamellata* Verseveldt and Tursch, 1979 features a “thin, plate-like funnel-wall” (see p. 143, plate 6), thus also resembling the colonies of *S. mesophotica* (this study: Figs 1, 4). For comparison, in the current study we re-examined the sclerites of the holotype of *S. lamellata* (RMNH Coel no. 12864), and SEM images are provided (Figs 6, 7). The surface layer of the polypary contains clubs, up to 0.16 mm long, some featuring a central wart comprised of a markedly spiny head (Fig. 6A). The polyps have point sclerites with head either poorly developed or distinct, up to 0.12 mm long (Fig. 6B), along with longer ones, up to 0.23 mm (Fig. 6C). There are no collaret or tentacle sclerites. The lack of collaret and tentacle sclerites in this specimen was observed previously (McFadden et al. 2009), and is probably caused by storing the specimen in formalin as many sclerites showed damage. A specimen from Palau identified as *S. lamellata* was also examined by McFadden et al. (2009), and that specimen showed collaret, point and tentacle sclerites (in situ photograph of that specimen available in WoRMS). The surface of the polypary contains some pointed spindles, up to 0.40 mm long (Fig. 6D). The longer ones might be transitional forms to the internal spindles of the polypary (Fig. 6E). Neither the point sclerites nor the spindles of the polypary were mentioned in the original description (see Verseveldt and Tursch 1979). The surface of the colony base contains clubs similar to those of the polypary, measuring up to 0.18 mm long, but some feature a wider head (Fig. 7A).

The interior of the polypary has almost straight spindles or slightly bent ones, up to 4.8 mm long (Fig. 6E), ornamented with simple low conical warts (Fig. 6F). The interior of the colony base has slightly bent or almost straight spindles, up to 6.3 mm long (Fig. 7B, C), with simple warts (Fig. 7D).

A comparison of *Sinularia lamellata* with *S. mesophotica* reveals that the club heads of the two species are quite different, as the former feature terminal prominences consisting of closely-packed, pointed, thin spikes (see also Verseveldt and Tursch 1979), while in the latter they are mostly truncate. Furthermore, *S. lamellata* possesses spindles on the surface of the polypary, where none exist in *S. mesophotica*. The polyp sclerites of both species markedly differ, with *S. mesophotica* having only collaret and tentacle sclerites while *S. lamellata* has points as well as collaret and tentacle sclerites. Similarly, the coenenchymal spindles of *S. lamellata* are longer and their ornamentation differs from that of *S. mesophotica*. It is thus evident that *S. mesophotica* differs morphologically from both *S. brassica* and *S. lamellata*, the two previously described *Sinularia* species that possess a similar funnel-shaped polypary.

*Sinularia frondosa* Verseveldt, 1978 also has somewhat similar sclerites, but this species does not have the funnel-shaped polypary. Moreover, McFadden et al. (2009) re-examined the type of *S. frondosa* and found that it shares the morphological characters of clade 5B, like *S. lamellata*.

The phylogenetic analyses also support the genetic distinction of *Sinularia mesophotica* sp. n. from *S. brassica*, *S. lamellata*, and all other species of *Sinularia* for which molecular data are available (Fig. 5). *Sinularia brassica*, including the morphologically and genetically distinct *dura* form with a funnel shape, is phylogenetically distinct from all other *Sinularia* species, comprising the previously described clade 1 (McFadden et al. 2009), whereas reference specimens identified morphologically as *S. lamellata* belong to clade 5B. In contrast, *S. mesophotica* occupies a unique phylogenetic position outside of any of the five recognized clades to which all other known *Sinularia* species belong, and can be considered to represent a sixth, phylogenetically distinct clade (Fig. 5).

The five previously recognized clades of *Sinularia*, as well as distinct subclades within the two large clades 4 and 5, can be distinguished morphologically based on a suite of four primary morphological characters. These include the presence of sclerites in the (a) tentacles, (b) collaret, and (c) points regions of the polyp, as well as (d) the shape of the club sclerites in the colony surface tissues (McFadden et al. 2009). For example, clade 1 (*S. brassica*) has scales in the tentacles but lacks a collaret and points, and the surface club sclerites are characterized by very wide heads of unique form. In contrast, species belonging to clade 5B (which includes *S. lamellata*) have sclerites in the tentacles, a collaret and points, and surface clubs with a distinct central wart. Clade 6 (*S. mesophotica* sp. n.) can be distinguished from all other clades by the presence of rods in the tentacles, a collaret but no points, and surface clubs with a central wart that may be indistinct or absent. It is evident that *S. mesophotica* differs both morphologically and genetically from all other congeners, and is thus justified to be a new species.

## Supplementary Material

XML Treatment for Sinularia mesophotica
